# The quick reference card “Storage of urinary EVs” – A practical guideline tool for research and clinical laboratories

**DOI:** 10.1002/jev2.12286

**Published:** 2023-03-14

**Authors:** Martin E. van Royen, Carolina Soekmadji, Cristina Grange, Jason P. Webber, Tobias Tertel, Marvin Droste, Anja Buescher, Bernd Giebel, Guido W. Jenster, Alicia Llorente, Charles J. Blijdorp, Dylan Burger, Uta Erdbrügger, Elena S. Martens‐Uzunova

**Affiliations:** ^1^ Department of Pathology Erasmus MC Cancer Institute Erasmus University Medical Center Rotterdam The Netherlands; ^2^ School of Biomedical Sciences Faculty of Medicine University of Queensland Brisbane Australia; ^3^ Department of Medical Sciences University of Turin Turin Italy; ^4^ Institute of Life Science Swansea University Medical School Swansea University Swansea UK; ^5^ Institute for Transfusion Medicine University Hospital Essen University of Duisburg‐Essen North Rhine‐Westphalia Germany; ^6^ Department of Pediatrics II (Pediatric Nephrology) University Hospital Essen University of Duisburg‐Essen North Rhine‐Westphalia Germany; ^7^ Department of Molecular Cell Biology Oslo University Hospital Oslo Norway; ^8^ Department for Mechanical Electronics and Chemical Engineering Oslo Metropolitan University Oslo Norway; ^9^ Department of Internal Medicine Division of Nephrology and Transplantation Erasmus University Medical Center Rotterdam The Netherlands; ^10^ Kidney Research Centre Ottawa Hospital Research Institute University of Ottawa Canada; ^11^ Department of Medicine Division of Nephrology University of Virginia Charlottesville Virginia USA; ^12^ Department of Urology Erasmus MC Cancer Institute Erasmus University Medical Center Rotterdam The Netherlands

Dear Editor,

The high diagnostic potential of urinary extracellular vesicles (uEVs) for urogenital disease has been recognized for more than a decade. This is emphasized by the identification of different molecular biomarkers (i.e. protein, mRNA, miRNA, lipids and metabolites) in uEV preparations that may assist the clinical management of prostate, bladder, and renal cancer (Junker et al., 2016). uEV biomarkers for other pathologies like acute and chronic kidney disease of various etiologies, cystic and tubule‐interstitial disease, or for kidney transplantation are also under active investigation (Grange & Bussolati, 2022).

Apart from the growing need for validation studies, the translational potential of uEV biomarkers is hampered by several biological factors. Such factors include the diverse cellular origins of uEVs throughout the renal and urogenital tract, but also the dynamic molecular composition of urine due to hydration status, diet, salt regulation, exercise, and circadian rhythm. In addition to these inherent factors, the reproducibility of uEV analysis is also strongly influenced by logistic variables like the differences in the time of sampling or the preanalytical procedures for handling of urine samples (Erdbrügger et al., 2021).

The general reporting recommendations for EV sample processing and analysis are covered in detail in the Minimal Information for Studies of Extracellular Vesicles (MISEV 2018) position paper (Thery et al., 2018). However, a community consensus on best methodological practices that is tailored to the biofluid‐specific characteristics and requirements is of particular importance for the success of preclinical and clinical studies on biomarker discovery, validation and future use in clinical decision making. To address this need in uEVs research, the Urine Task Force of the Rigor and Standardization Subcommittee of the *International Society for Extracellular Vesicles* (*ISEV*) published a position paper summarizing the current *state of the art* and listing detailed recommendations for improved rigor, reproducibility and inter‐operability in uEV research (Erdbrügger et al., 2021).

To support the implementation of the published recommendations, and enhance their application in daily research practices, here we provide a Quick Reference Card on *STORAGE of urinary EVs* (Figures 1 and 2, Supplementary File 1). The Quick Reference Card does not substitute a uEVs protocol for storage, isolation or processing but it summarizes the expert community consensus recommendations on the most critical factors affecting storage of fresh or biobank urine and uEVs samples as discussed in the uEV position paper (Erdbrügger et al., 2021). The Card is organized according to six critical stages: Biobanking, Storage of urine prior to processing, Preprocessing, Storage of urinary supernatant and uEVs, Defrosting, and Transportation. Evidence level and reporting priority for each stage are color‐coded in accordance to the findings as described in the ISEV uEVs position paper (Erdbrügger et al., 2021) and according to the MISEV 2018 guidelines (Thery et al., 2018). The Card is intended as an easily accessible guideline tool that can be used during study planning and manuscript preparation, but also as a “bench top” reference during everyday laboratory work.

**FIGURE 1:**
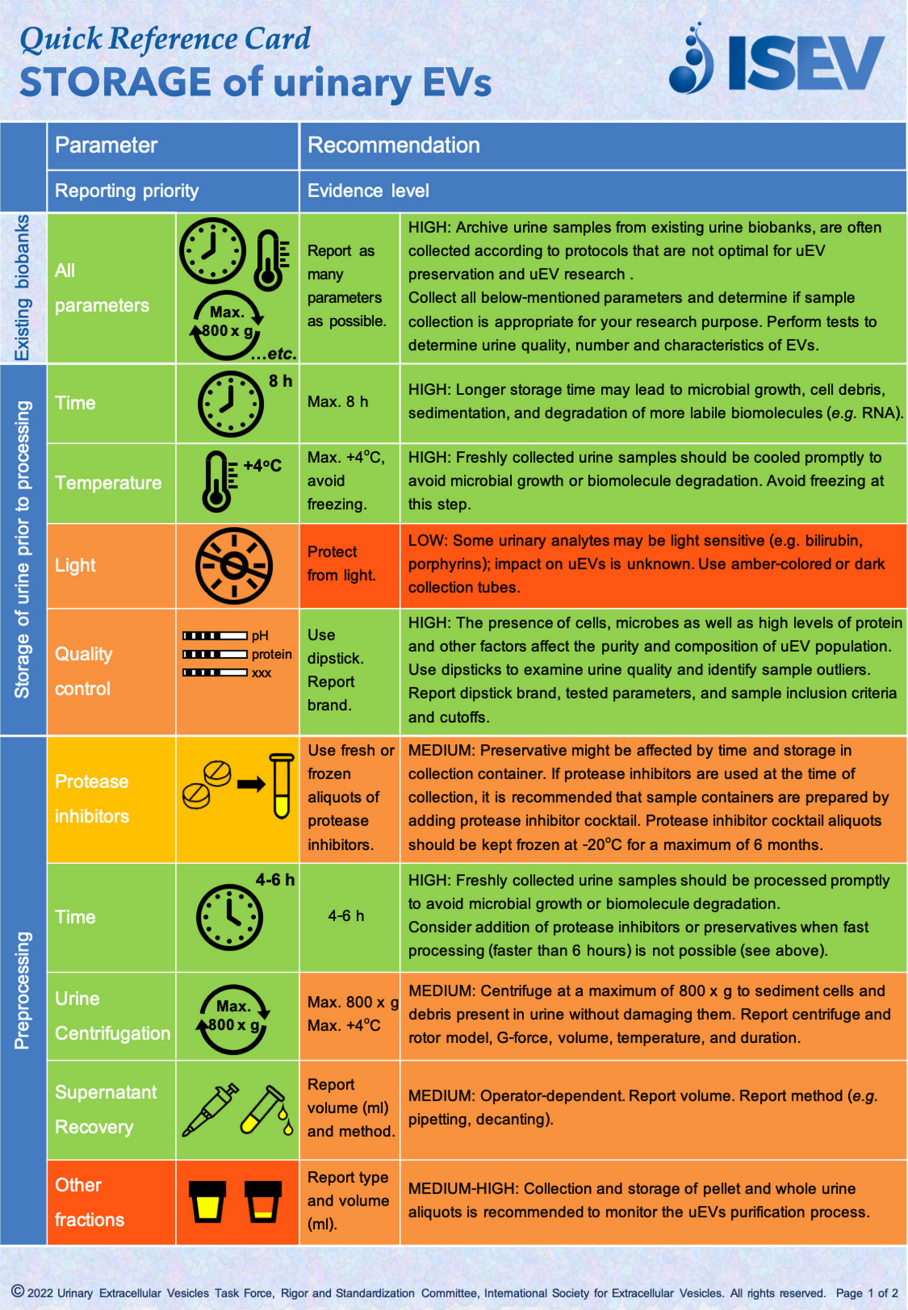
Quick Reference Card “Storage of urinary EVs”, page 1 *Storage of urine prior to processing* and *Pre‐processing steps*. Priority and Evidence levels are as reported in (Erdbrügger et al., 2021) and represent expert consensus opinion of the current level of confidence that the parameter is a variable to consider during sample biobanking and data analysis and interpretation.

**FIGURE 2:**
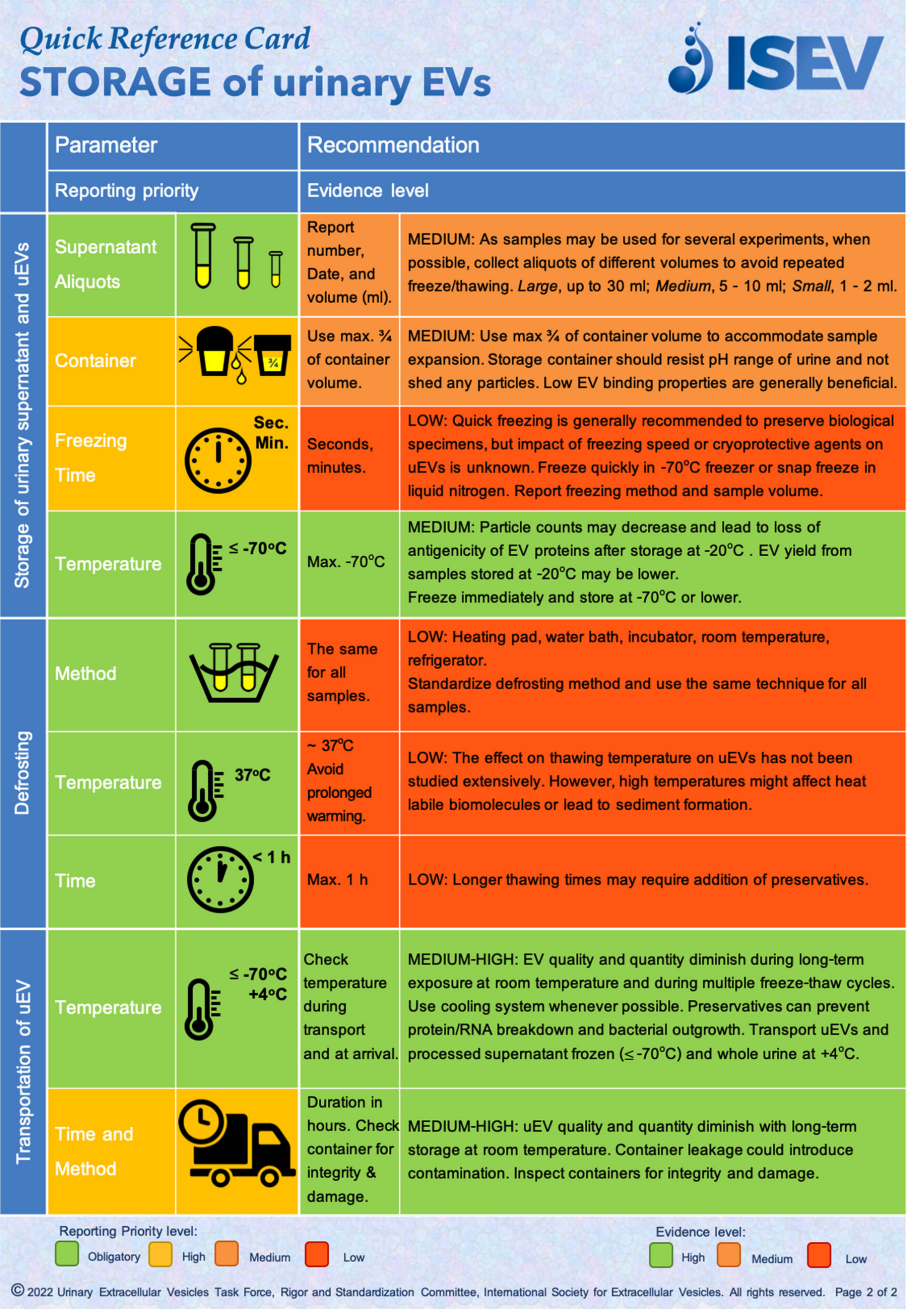
Quick Reference Card “Storage of urinary EVs”, page 2 *Storage of urinary supernatant and uEVs, Defrosting*, and *Transportation of uEVs*. Priority and Evidence levels are as reported in (Erdbrügger et al., 2021) and represent expert consensus opinion of the current level of confidence that the parameter is a variable to consider during sample biobanking and data analysis and interpretation.

To conclude, we present a novel format of communication for EV study guidelines and recommendations that can also be applied to other topics within, but importantly also outside the field of urinary EVs. Ultimately, by using this format, we endeavor to enhance adherence to pre‐analytical best practice guidelines in order to promote reproducibility and, above all, the translational potential of uEV studies.

## CONFLICTS OF INTEREST

The authors report no conflict of interest.

## AUTHOR CONTRIBUTIONS

Conceptualization: M.v.R., C.S., C.G., J.W., T.T., M.D., A.B., B.G., A.L., C.B., D.B., U.E., E.M.U. Writing, original draft preparation: M.v.R., E.M.U.; Writing, review and editing: M.v.R., C.S., C.G., J.W., T.T., M.D., A.B., B.G., A.L., C.B., D.B., U.E., E.M.U.

All authors have read and agreed to the published version of the manuscript.

## Supporting information

Supplementary File 1. Quick Reference Card: Storage of Urinary EVs
