# Supplementary material for: The quick reference card “Storage of urinary EVs” – A practical guideline tool for research and clinical laboratories
Source: J Extracell Vesicles. 2023 Mar 14;12(3):12286. doi: 10.1002/jev2.12286 (PMC10011888; doi:10.1002/jev2.12286)
Supplement: Supplementary file 1 — Quick Reference Card: Storage of Urinary EVs [file JEV2-12-12286-s001.pdf]

Quick Reference Card

STORAGE of urinary EVs

ISEV

	Parameter<br>Reporting priority		Recommendation	Evidence level
Existing biobanks	All parameters	Max. 800 x g …etc.	Report as many parameters as possible.	HIGH: Archive urine samples from existing urine biobanks, are often collected according to protocols that are not optimal for uEV preservation and uEV research . Collect all below-mentioned parameters and determine if sample collection is appropriate for your research purpose. Perform tests to determine urine quality, number and characteristics of EVs.
Storage of urine prior to processing	Time	8 h	Max. 8 h	HIGH: Longer storage time may lead to microbial growth, cell debris, sedimentation, and degradation of more labile biomolecules (*e.g.* RNA).
Storage of urine prior to processing	Temperature	+4°C	Max. +4°C, avoid freezing.	HIGH: Freshly collected urine samples should be cooled promptly to avoid microbial growth or biomolecule degradation. Avoid freezing at this step.
Storage of urine prior to processing	Light		Protect from light.	LOW: Some urinary analytes may be light sensitive (e.g. bilirubin, porphyrins); impact on uEVs is unknown. Use amber-colored or dark collection tubes.
Storage of urine prior to processing	Quality control	pH<br>protein<br>xxx	Use dipstick. Report brand.	HIGH: The presence of cells, microbes as well as high levels of protein and other factors affect the purity and composition of uEV population. Use dipsticks to examine urine quality and identify sample outliers. Report dipstick brand, tested parameters, and sample inclusion criteria and cutoffs.
Preprocessing	Protease inhibitors		Use fresh or frozen aliquots of protease inhibitors.	MEDIUM: Preservative might be affected by time and storage in collection container. If protease inhibitors are used at the time of collection, it is recommended that sample containers are prepared by adding protease inhibitor cocktail. Protease inhibitor cocktail aliquots should be kept frozen at -20°C for a maximum of 6 months.
Preprocessing	Time	4-6 h	4-6 h	HIGH: Freshly collected urine samples should be processed promptly to avoid microbial growth or biomolecule degradation. Consider addition of protease inhibitors or preservatives when fast processing (faster than 6 hours) is not possible (see above).
Preprocessing	Urine Centrifugation	Max. 800 x g	Max. 800 x g<br>Max. +4°C	MEDIUM: Centrifuge at a maximum of 800 x g to sediment cells and debris present in urine without damaging them. Report centrifuge and rotor model, G-force, volume, temperature, and duration.
Preprocessing	Supernatant Recovery		Report volume (ml) and method.	MEDIUM: Operator-dependent. Report volume. Report method (*e.g.* pipetting, decanting).
Preprocessing	Other fractions		Report type and volume (ml).	MEDIUM-HIGH: Collection and storage of pellet and whole urine aliquots is recommended to monitor the uEVs purification process.

© 2022 Urinary Extracellular Vesicles Task Force, Rigor and Standardization Committee, International Society for Extracellular Vesicles. All rights reserved.

Quick Reference Card

STORAGE of urinary EVs

	Parameter		Recommendation	
	Reporting priority		Evidence level	
Storage of urinary supernatant and uEVs	Supernatant Aliquots		Report number, Date, and volume (ml).	MEDIUM: As samples may be used for several experiments, when possible, collect aliquots of different volumes to avoid repeated freeze/thawing. *Large*, up to 30 ml; *Medium*, 5 - 10 ml; *Small*, 1 - 2 ml.
	Container	¾	Use max. ¾ of container volume.	MEDIUM: Use max ¾ of container volume to accommodate sample expansion. Storage container should resist pH range of urine and not shed any particles. Low EV binding properties are generally beneficial.
	Freezing Time	Sec. Min.	Seconds, minutes.	LOW: Quick freezing is generally recommended to preserve biological specimens, but impact of freezing speed or cryoprotective agents on uEVs is unknown. Freeze quickly in -70°C freezer or snap freeze in liquid nitrogen. Report freezing method and sample volume.
	Temperature	≤ -70°C	Max. -70°C	MEDIUM: Particle counts may decrease and lead to loss of antigenicity of EV proteins after storage at -20°C . EV yield from samples stored at -20°C may be lower.<br>Freeze immediately and store at -70°C or lower.
Defrosting	Method		The same for all samples.	LOW: Heating pad, water bath, incubator, room temperature, refrigerator.<br>Standardize defrosting method and use the same technique for all samples.
	Temperature	37°C	~ 37°C Avoid prolonged warming.	LOW: The effect on thawing temperature on uEVs has not been studied extensively. However, high temperatures might affect heat labile biomolecules or lead to sediment formation.
	Time	< 1 h	Max. 1 h	LOW: Longer thawing times may require addition of preservatives.
Transportation of uEV	Temperature	≤ -70°C +4°C	Check temperature during transport and at arrival.	MEDIUM-HIGH: EV quality and quantity diminish during long-term exposure at room temperature and during multiple freeze-thaw cycles. Use cooling system whenever possible. Preservatives can prevent protein/RNA breakdown and bacterial outgrowth. Transport uEVs and processed supernatant frozen (≤-70°C) and whole urine at +4°C.
	Time and Method		Duration in hours. Check container for integrity & damage.	MEDIUM-HIGH: uEV quality and quantity diminish with long-term storage at room temperature. Container leakage could introduce contamination. Inspect containers for integrity and damage.

Reporting Priority level: Obligatory, High, Medium, Low

Evidence level: High, Medium, Low

© 2022 Urinary Extracellular Vesicles Task Force, Rigor and Standardization Committee, International Society for Extracellular Vesicles. All rights reserved.
